# The reliability of toe systolic pressure and the toe brachial index in patients with diabetes

**DOI:** 10.1186/1757-1146-4-S1-P52

**Published:** 2011-05-20

**Authors:** Mary Romanos, Anita Raspovic, Byron Perrin

**Affiliations:** 1 Department of Podiatry and Musculoskeletal Research Centre, Faculty of Health Sciences, La Trobe University, Bundoora, Victoria, 3086, Australia; 2 La Trobe Rural Health School and Musculoskeletal Research Centre, Faculty of Health Sciences, La Trobe University, Bendigo, Victoria, 3552, Australia

## Background

The ankle brachial index is a useful clinical test for establishing blood supply to the foot. However, there are limitations to this method when conducted on people with diabetes. As an alternative to the ankle brachial index, measuring toe systolic pressures and the toe brachial index have been recommended to assess the arterial blood supply to the foot. This study aimed to determine the intra and inter-rater reliability of the measurement of toe systolic pressure and the toe brachial index in patients with diabetes using a manual sphygmomanometer and photoplethysmography unit.

## Methods

This was a repeated measures, reliability study. Three raters measured toe systolic pressure and the toe brachial index in thirty participants with diabetes. Measurement sessions occurred on two occasions, one week apart, using a manual photoplethysmography unit (Hadeco Smartdop 45) and a standardised measurement protocol. Intra-class correlation coefficients and 95% limits of agreement (LOA) were calculated.

## Results

The mean intra-class correlation for intra-rater reliability for toe systolic pressures was 0.87 (95% LOA: -25.97 to 26.06 mmHg) and the mean intra-class correlation for Toe Brachial Indices was 0.75 (95% LOA: -0.22 to 0.28). The intra-class correlation for inter-rater reliability was 0.88 for toe systolic pressures (95% LOA: -22.91 to 29.17.mmHg) and 0.77 for toe brachial indices (95% LOA: -0.21 to 0.22).

## Conclusions

Despite the reasonable intra-class correlation results, the range of error was broad. This potentially clinically significant margin of error raises questions about the reliability of using a manual sphygmomanometer and photoplethysmography to measure toe systolic pressure and toe brachial index. When assessing patients with peripheral arterial occlusive disease, it is important to consider all other non-invasive vascular assessment options.

